# *De novo* whole genome assembly of the globally invasive green shore crab *Carcinus maenas* (Linnaeus, 1758) via long-read Oxford Nanopore MinION sequencing

**DOI:** 10.1093/jhered/esaf085

**Published:** 2025-10-22

**Authors:** Jolanda K Brons, Thomas Hackl, Riccardo Iacovelli, Kristina Haslinger, Sebastian Lequime, Sancia E T van der Meij

**Affiliations:** Groningen Institute for Evolutionary Life Sciences, University of Groningen, Groningen, The Netherlands; Groningen Institute for Evolutionary Life Sciences, University of Groningen, Groningen, The Netherlands; Groningen Research Institute of Pharmacy, University of Groningen, Groningen, The Netherlands; VTT Technical Research Centre of Finland, Espoo, Finland; Groningen Research Institute of Pharmacy, University of Groningen, Groningen, The Netherlands; Groningen Institute for Evolutionary Life Sciences, University of Groningen, Groningen, The Netherlands; Groningen Institute for Evolutionary Life Sciences, University of Groningen, Groningen, The Netherlands; Naturalis Biodiversity Center, Leiden, The Netherlands

**Keywords:** Brachyura, Carcinidae, European green crab, invasion genomics

## Abstract

Invasive species are reshaping aquatic ecosystems worldwide at an accelerating pace, with profound ecological and economic impacts. Many crustacean species have demonstrated invasive potential or are already well-established invaders. The green shore crab, *Carcinus maenas*, native to Europe and North Africa, is one of the most successful global marine invaders and is now present on six continents. Although the role of genomics in invasion science is increasingly recognized, genomic resources for brachyuran crabs remain limited, including the notable absence of a reference genome for *C. maenas*. Here we report on a de novo whole genome assembly of *C. maenas* via long–read Oxford Nanopore Technology sequencing. The assembly spans 1.09 Gbp across 21 887 scaffolds (NG50 = 13 Mbp) with a BUSCO completeness of 98.4%, providing a high-quality resource for future genomic analyses. We provide a detailed protocol for obtaining high-quality DNA to successfully sequence brachyuran crabs using a long-read approach. This new resource expands available genomic data for the species–rich infraorder Brachyura, and provides a valuable foundation for understanding the genetic factors underlying the global invasion success of *C. maenas*, supporting future research in marine invasion genomics.

## Introduction

Invasive species are transforming marine habitats worldwide (Molnar et al. 2008), and the rate at which species occur outside their native ranges has increased considerably over the past few centuries. This increase does not show any sign of saturation, indicating that current efforts to mitigate invasions are inadequate to keep up with the accelerating impacts of globalization (Seebens et al. 2017). While the drivers of biological invasion are increasingly global in nature, the impacts of invasions are mainly observed at local scales (Molnar et al. 2008). Some non-native species might provide benefits to an ecosystem (Schlaepfer 2018), while others can cause severe ecological or economic damage, making them invasive (Simberloff et al. 2013). The negative impacts of invasive species range from habitat destruction and disease transmission to displacement or even extinction of native species (Molnar et al. 2008).

Invasive species often possess traits linked to invasion success, including broad environmental tolerance, high reproductive output (r-strategists), phenotypic plasticity, effective dispersal, and competitive advantages (Davidson et al. 2011; Weir & Salice 2011). Additionally, invasive species may gain an additional advantage by escaping natural enemies, although this effect should not be overstated (Colautti et al. 2004). The success of many invasive species may rely more on their capacity to adapt through natural selection than on general physiological tolerance or plasticity alone (Lee 2002). Whole-genome data are invaluable for advancing the field of invasion genomics (Jaspers et al. 2021) and offer powerful tools to enhance our understanding of biological invasions in aquatic systems (Tepolt 2015; Bourne et al. 2018; North et al. 2021). For example, comparative whole-genome sequencing in crustaceans demonstrated that many expanded gene families are involved in the environmental tolerance of the red swamp crayfish *Procambarus clarkii* (Girard, 1852) (Xu et al. 2021), whereas the invasion potential of the Chinese mitten crab *Eriocheir sinensis* (H. Milne Edwards, 1853) is largely attributed to its strong osmoregulatory capacity and high fertility (Cui et al. 2021). Despite their potential, these resources so far remain underexploited in efforts to predict invasive potential (Kołodziejczyk et al. 2025).

The list of the 100 most notorious globally invasive species in the Global Invasive Species Database, compiled by Lowe et al. (2000), includes nine aquatic invertebrates. Whole genome data is currently available for six of these species. Three out of six species are molluscs: the golden apple snail (*Pomacea canaliculata* Lamarck, 1822) (Lu et al. 2024), the Mediterranean mussel (*Mytilus galloprovincialis*; Lamarck, 1819) (Han et al. 2024), and the zebra mussel (*Dreissena polymorpha* Pallas, 1771) (McCartney et al. 2022). Whole genome data is furthermore available for an echinoderm, the Northern Pacific seastar (*Asterias amurensis* Lutken, 1871) (Wang et al. 2023); a ctenophore, *Mnemiopsis leidyi* (A. Agassiz, 1865) (Ryan et al. 2013); and a crustacean, the Chinese mitten crab (*E. sinensis*) (Cui et al. 2021). Whole genome data is so far missing for three invasive aquatic species: the fishhook waterflea (*Cercopagis pengoi* Ostroumov, 1891), the Asian clam (*Potamocorbula amurensis* Schrenck, 1861), and the green shore crab (*Carcinus maenas* Linneaus, 1758).

*Carcinus maenas* is native to Europe and northwest Africa. It is one of the world’s most widespread invasive marine species, now recorded from six continents (Carlton and Cohen 2003; GBIF 2025; Supplementary Fig. 1). Despite its global distribution and ecological impact, no reference genome is currently available for this species. As a generalist predator with a broad diet, *C. maenas* poses serious threats to native biodiversity across its introduced range (Ens et al. 2022). Due to its adaptability and resilience, *C. maenas* is likely to continue expanding into new ecosystems and may trigger secondary invasion waves in areas it already inhabits (Jeffery et al. 2017; Frederich and Lancaster 2024—and references therein). The lack of genomic data for *C. maenas* highlights a wider gap in genomic resources for decapod crustaceans (Yuan et al. 2023). Obtaining whole decapod genomes is particularly challenging due to their large size and high content of repetitive DNA (Van Quyen et al. 2020; Cui et al. 2021; Rutz et al. 2023). The infraorder Brachyura (true crabs) accounts for almost half of the total diversity in Decapoda, and this group has not only successfully conquered the marine realm, from the deep sea to the intertidal, but is also successful in a multitude of freshwater and terrestrial environments (De Grave et al. 2023). Despite the diversity of this group, with close to 8,000 species, and the presence of several notorious invaders and economically important species, only 10 reference genomes across five families are available for brachyuran crabs (NCBI 2025), with seven more on the way (Wang et al. 2025).

In this study, we present a whole genome assembly of *C. maenas* (family Carcinidae MacLeay 1838), based on a specimen from within its native range, using long-read sequencing (Oxford Nanopore Technologies (ONT), Oxford, UK). Nanopore technology is a third-generation sequencing method that produces long reads of consistent quality and is recognized as an affordable option for de novo sequencing, even for complex genomes (de Lannoy et al. 2017). Although this technology has significant potential to advance genomic research on non-model organisms like aquatic invertebrates, its application is often hindered by challenges in extracting and purifying genomic DNA from many species (Daniels et al. 2023). To address this, we provide a detailed, step-by-step protocol for obtaining sufficient DNA for Nanopore sequencing and genome assembly, along with a practical guide to sequencing brachyuran crabs using a MinION.

## Material and methods

### Specimen collection

Preliminary test runs were performed using an adult male *C. maenas* collected on 26 April 2023 from the marina of Schiermonnikoog, the Netherlands (53.470427, 6.166608). The specimen (Supplementary Fig. 2A) was preserved in 96% ethanol and stored at −20°C. Data from these initial runs contributed to the final genome assembly. The whole genome assembly was based on a second adult male *C. maenas* (Supplementary Fig. 2B), collected on 11 April 2024 from the harbor of Lauwersoog, the Netherlands (53.410545, 6.208381). This specimen was preserved in DESS solution containing 20% dimethyl sulfoxide, 0.25 M ethylenediaminetetraacetic acid (EDTA), and saturated sodium chloride (NaCl), then stored at −20°C until DNA extraction (Oosting et al. 2020). Both crabs remain vouchered at the University of Groningen.

### Preliminary MinION runs and method optimization

During initial MinION test runs using muscle tissue from the Schiermonnikoog specimen, consistent pore blockage occurred approximately 2 h into sequencing. To address this, we tested two preservation fluids, optimized DNA extraction protocols, employed two short fragment removal (SFR) kits and a whole genome amplification (WGA) to eliminate potential contaminants responsible for the blockages.

First, we evaluated two different DNA extractions methods, the Modified Qiagen G/20 Tip DNA Extraction and Powersoil Pro DNA Extraction.

### Modified Qiagen G/20 tip DNA extraction

Approximately 400 mg of muscle tissue from the specimens’ pereiopods was finely minced. The tissue was suspended in 800 μl of 1x phosphate-buffered saline (PBS, Fisher Scientific, Hampton, USA) and centrifuged at 4,000 × g for 1 min at room temperature. The supernatant was discarded, and the washing step was repeated. The pellet was then resuspended in a solution of 16 μl RNase (Qiagen, Hilden, Germany) and 2 ml G2 buffer (Genomic DNA Buffer Set, Qiagen, Hilden, Germany). Subsequently, 250 μl of proteinase K (>600 mAU/ml, Qiagen, Hilden, Germany) was added, and the mixture was incubated at 50°C for 18 h.

After incubation, the mixture was centrifuged at 4,000 × g for 5 min at room temperature. The supernatant was transferred to a new tube, and 1 ml of InhibitEX buffer (Qiagen, Hilden, Germany) was added to remove contaminants (Boughattas et al. 2021). The mixture was incubated at room temperature for 5 min and then centrifuged again at 4,000 × g for 5 min. The resulting supernatant was used for genomic DNA extraction.

Genomic DNA from *C. maenas* was extracted using the “Isolation of Genomic DNA from Blood, Cultured Cells, Tissue, Yeast, or Bacteria Using Genomic-tips” protocol outlined in the Qiagen Genomic DNA Handbook, with the following slight modifications. A Qiagen genomic-tip 20/G (Qiagen Genomic-tip 20/G Kit, Qiagen, Hilden, Germany) was equilibrated with 1.0 ml of QBT buffer (Genomic DNA Buffer Set) and allowed to empty via gravity flow. The *C. maenas* sample was then applied to the prepared genomic-tip 20/G. After the sample passed through, the genomic tip was washed three times with 1.0 ml of QC buffer (Genomic DNA Buffer Set).

The genomic DNA was eluted with 2.0 ml of pre-warmed (50°C) QF buffer (Genomic DNA Buffer Set) and precipitated by adding 0.7 volumes (1.4 ml) of room-temperature (15°C to 25°C) isopropanol (Sigma-Aldrich, Saint Louis, USA). The mixture was gently inverted several times and incubated at room temperature for 5 min. It was then centrifuged at 5,000 × g for 15 min at 4°C. The supernatant was discarded, and the resulting pellet was washed with 1 ml of cold 70% ethanol (4°C) and centrifuged again at 5,000 × g for 10 min at 4°C. After removing the supernatant, the pellet was air-dried for 10 min and resuspended in 60 μl of pre-warmed (50°C) Tris-EDTA (TE) buffer (Sigma-Aldrich, Saint Louis, USA). The resuspension was incubated at 37°C for 1 h. DNA concentrations were measured using a Qubit Flex Fluorometer (Fisher Scientific, Waltham, USA). The A260/280 and A260/230 ratios were also assessed using a NanoDrop spectrophotometer (Thermo Fisher Scientific, Waltham, USA).

### Powersoil pro DNA extraction

The crab specimen was subsampled again, and approximately 400 mg of pereiopod muscle tissue was finely chopped. The tissue was combined with 800 μl of 1x PBS (Fisher Scientific, Hampton, USA) and centrifuged at 4,000 × g for 1 min at room temperature. The supernatant was discarded, and the washing step was repeated twice. DNA extraction was performed using the Powersoil Pro DNA Extraction Kit (Qiagen, Hilden, Germany) according to the manufacturer’s instructions, with the following modifications: 0.25 g of Zirconia beads (Biospec Products, Bartlesville, USA) were added, and the vortexing step (step 2, 10 min) was replaced with two 60-s bead-beating cycles at 6.0 m/s using a FastPrep-24™ 5G bead beater (MP Biomedicals, Santa Ana, USA). DNA quality and quantity were assessed as described above.

Next, an SFR step was applied to both DNA extraction methods to enhance the mean read length and investigate pore blockage caused by small reads.

### SFR

This process removed short DNA fragments up to 10 kb (SFR-A) or 25 kb (SFR-B). The SFR-A solution was prepared with 4% PVP 360,000, 1.2 M KCl, and 20 mM Tris–HCl (pH 8), while the SFR-B solution contained 3% PVP 360000, 1.2 M NaCl, and 20 mM Tris–HCl (pH 8) (Jones et al. 2021). Sixty μl of the *C. maenas* genomic DNA sample was mixed with 60 μl of either SFR-A or SFR-B solution, and the mixture was homogenized by gently tapping the tube. For SFR-B, the mixture was first incubated for 1 h at 50°C. Both SFR-A and SFR-B samples were then centrifuged at 10,000 × g for 30 min at room temperature. The supernatant was carefully removed, and 200 μl of cold 70% ethanol was added, followed by centrifugation at 10,000 × g for 2 min at room temperature. This ethanol-washing step was repeated three times. The pellet was subsequently dried for 10 min at 50°C and resuspended in 60 μl of pre-warmed (37°C) TE buffer for 20 min at 37°C. DNA quality and quantity were assessed as described above.

As the extraction/purification combinations did not resolve the pore blockage, whole-genome purification was performed as a final attempt.

### WGA

To address persistent pore blockage in the MinION flow cell during sequencing, several approaches were tested to remove potential contaminants, including alternative DNA extraction methods, incorporation of an InhibitEX step, and SFR (as previously described). Despite these efforts, the blockage issue persisted. WGA was therefore performed on the modified G/20 tip DNA extraction + SFR-A sample using the REPLI-g Mini Kit (Qiagen, Hilden, Germany), following the manufacturer’s instructions, in an effort to eliminate the source of the blockage. DNA concentrations and purity ratios were again assessed using a Qubit Flex Fluorometer and a NanoDrop spectrophotometer.

### Library preparation and MinION sequencing

Genomic DNA libraries for *C. maenas* were constructed from DNA using the aforementioned extraction/purification combinations and WGA approach (see Supplementary Table 1 for details), with library preparation performed using the SQK-LSK114 Ligation Sequencing Kit V14 (ONT). Library preparation started with 1 μg of *C. maenas* DNA, quantified using a Qubit Flex Fluorometer. Following the “Ligation Sequencing gDNA V14” protocol provided by ONT, the process included a DNA repair and end-prep, adapter ligation, and clean-up step. DNA concentrations were measured after each step using the Qubit Flex Fluorometer. The sequencing flow cell (R10.4.1, FLO-MIN114, ONT) was primed and loaded with 20 to 60 ng of *C. maenas* library per run (Supplementary Table 1). Sequencing was performed on a MinION device (ONT), basecalling and demultiplexing were performed with MinKNOW/Dorado at “super-accurate basecalling, 400 bps”.

### Pore blockage and sequencing optimization

Both the modified G/20 tip and Powersoil Pro DNA extraction methods, combined with either SFR-A or SFR-B or the WGA sample (modified G/20 tip DNA extraction + SFR-A kit), produced sufficient DNA for sequencing. The SFR kits (SFR-A and SFR-B) effectively reduced the number of small reads in the samples. However, none of the tested approaches, including WGA, were able to prevent pore blockage, which consistently occurred approximately 2 h into sequencing.

Interestingly, a pore scan revealed that some pores reopened after blockage, and a substantial increase in active pores was observed following the use of the Flow Cell Wash Kit (ONT), which contains DNase. This suggests that pore blockage may not be caused by contaminants, but by structural properties of the DNA itself. To mitigate blockage, we implemented a sequencing protocol that included a pore scan every 30 min during the run and a flow cell wash every 2 h (Supplementary Table 1).

### Genome assembly and curation

The read data was assembled with Flye v2.9.2 (Kolmogorov et al. 2019) specifying high-quality Nanopore reads as input (“--nano-hq”), and the assembly was polished with medaka v1.11.3 (https://github.com/nanoporetech/medaka).

Due to difficulties in the sequencing process and the resulting limited amount of sequencing data, we generated two initial assemblies. The first assembly used only the data from the Lauwersoog specimen, while the second combined data from this specimen with an additional 10% of reads from the Schiermonnikoog specimen obtained during the test phase. Although combining data from multiple specimens is not generally recommended, since it can introduce small sequence and structural variations that may negatively impact assembly quality, comparison of the statistics of the two assemblies indicated that the combined assembly had better contiguity and completeness. Based on this, we chose to proceed with the combined assembly for all subsequent analyses.

Because a complete mitochondrial genome was not detectable in our draft assembly, we used a targeted approach to assemble it. Mitochondria of crabs have a lower GC content than the nuclear genome, and mitochondrial DNA is present in high abundance in muscle tissue. We therefore extracted reads from our data with a GC content below 35% and a minimum length of 10 kbp and generated an assembly from a 10% subsample of the resulting reads to adjust the coverage to a level suited for assembly. Using mitochondrial-encoded cytochrome c oxidase subunit I protein sequences obtained from Interpro (IPR000883) as a marker (Blum et al. 2025), we identified a mitochondrial candidate contig using diamond blastx v2.19 (Buchfink et al. 2021) in the target assembly. Whole-genome self-alignment of the candidate sequence revealed that its palindromic structure was an artifact of the assembly process. We pruned the duplicated region to obtain the final mitochondrial sequence. Using this curated sequence as a reference, we removed remaining mitochondrial fragments from the whole genome draft based on minimap2 v2.28 (Li 2018) mappings before adding the curated mitochondrial sequence to the whole genome assembly.

To optimize our assembly in terms of contiguity, we used homology–based reference scaffolding to organize smaller contigs into larger scaffolds. Specifically, we used the homology scaffolder RagTag (Alonge et al. 2022), leveraging the highly contiguous genome assembly of the mud crab *Scylla paramamosain* (Estampador 1950 as reference) (RefSeq accession: GCF_035594125.1).

### Assembly statistics and quality assessment

To assess potential contamination of the assembly, we employed two complementary approaches. First, we ran the deep learning classifier DeepMicroClass v1.0.3 (Hou et al. 2024) for a broad classification of sequences into eukaryotes, prokaryotes, and viruses. Second, we used MMSeq2 homology-based “easy-taxonomy” workflow (Mirdita et al. 2021) with UniRef50 (The UniProt Consortium 2025) as database to assign each contig a last common ancestor classification based on sequence homology.

To estimate assembly completeness, we used Compleasm v0.2.6 (Huang and Li 2023) with BUSCO (Tegenfeldt et al. 2025) marker gene sets and automatic lineage detection. To identify low-complexity regions within the sequences, we employed tantan v26 (Frith 2011). Additionally, we gathered basic assembly statistics—such as sequence length and base composition—using SeqKit (Shen et al. 2024).

## Results

To obtain high-quality, high-molecular-weight DNA suitable for long-read sequencing of brachyuran crabs, we evaluated two extraction protocols: the Modified Qiagen Genomic-tip 20/G (G/20) method and the DNeasy Powersoil Pro Kit. Each was tested in combination with either of two SFR kits (SFR-A or SFR-B). Despite yielding high-quality DNA in all cases, none of these extraction/purification combinations prevented Nanopore sequencing pore blockage, which consistently occurred approximately 2 h after run initiation. To address this, we applied WGA to a *C. maenas* sample extracted with the Modified G/20 method and SFR-A. However, this approach also failed to mitigate the pore blockage issue. While pore blockage remained a limiting factor, all extraction and purification strategies tested (including WGA) produced DNA of sufficient quality for successful sequencing. Each method generated high-quality reads, indicating that these protocols are suitable for sequencing brachyuran crabs with long-read platforms. To reduce pore loss and improve data yield, we incorporated periodic pore scans (every 30 min) and applied a flow cell wash after each run. This approach partially restored pore activity, enhancing overall sequencing throughput.

Based on existing measurements of *C. maenas* cellular DNA contents (1.24 pg and 1.07 pg; Gregory 2025), we estimated its genome size to be approximately 1.13 Gbp. We successfully generated 11.1 Gbp of *C. maenas* DNA sequence data (Lauwersoog specimen) using five MinION flow cells, achieving an average read N50 of 6.11 kbp. This dataset was supplemented with an additional 1.2 Gbp of data generated from the initial test sequencing runs on the Schiermonnikoog specimen (see Supplementary Table 1 for details). The whole genome of *C. maenas* was assembled de novo and polished, generating an initial contig assembly of 965 Mbp, comprising 34,569 contigs and an NG50 of 54 kbp. We further scaffolded our draft contigs based on homology to the mud crab (*S. paramamosain)* and curated the resulting scaffolds to ensure high quality (see Material and Methods). We obtained a final genome assembly of 1.09 Gbp in size, comprising 21,887 scaffolds and an NG50 of 13 Mbp (i.e. more than 50% of the genome is contained in scaffolds larger than 13 Mbp).

**Fig. 1:**
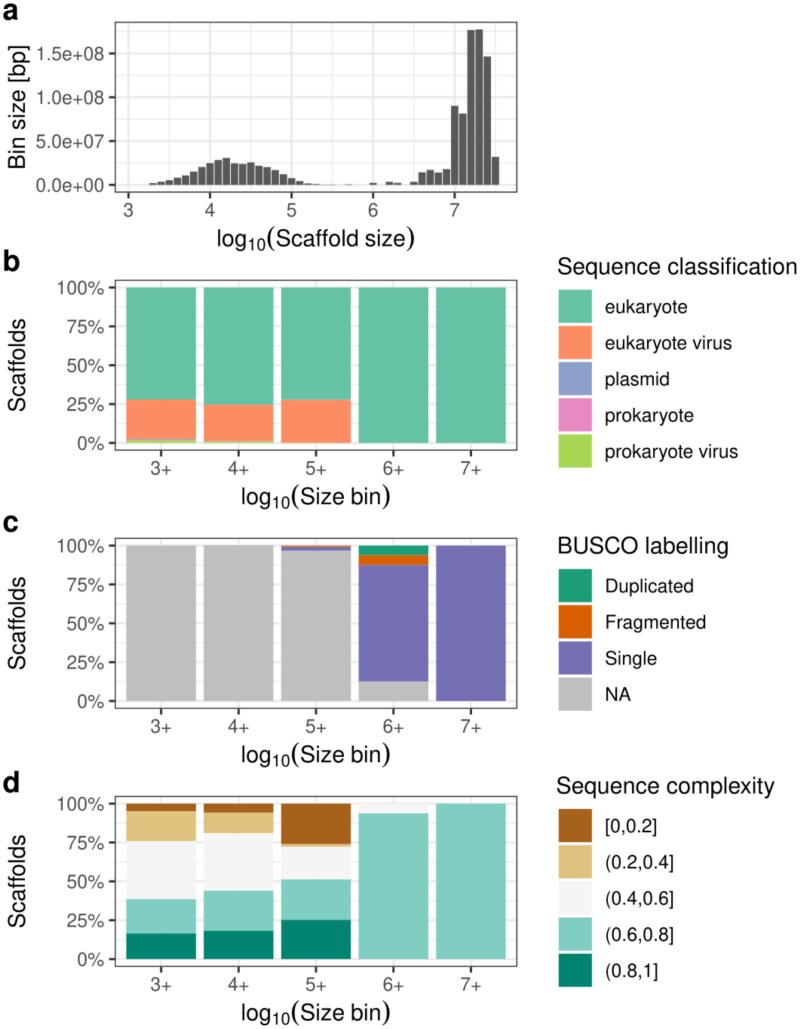
Overview of assembly composition and quality metrics. (a) Size distribution histogram of assembled contigs and scaffolds. (b) Broad classification profiles across contigs/scaffolds of different length bins (minimum sizes: 1 kb, 10 kb, 100 kb, 1 Mb, 10 Mb), with colors indicating the predicted source. (c) Distribution of universal eukaryotic marker genes (BUSCOs) across the same contigs/scaffolds length bins. (d) Sequence complexity profiles across contigs/scaffolds length bins with colors indicating the fraction of sequences identified as low complexity by the tantan algorithm.

A closer examination of the number and size distribution of the assembled sequences reveals a bimodal pattern (Fig. 1a). Approximately 80% of the genome is represented by chromosome-scale scaffolds, while the remainder consists predominantly of shorter sequences ranging from 1 to 100 kbp. This distribution is consistent with our reference–based scaffolding approach, where some contigs were either unplaced or placed ambiguously due to limited or conflicting homology information.

Assessment of contamination via two complementary approaches indicated negligible levels of sequences predicted to derive from sources other than the genuine *C. maenas* genome (Fig. 1b, Supplementary Table 2). Using broad deep-learning–based classification, scaffolds larger than 1 Mbp were exclusively classified as eukaryotic, while three-fourths of smaller scaffolds were classified as eukaryotic and one-fourth as eukaryotic viruses. In the majority, the latter sequences represent retrotransposons that are expected to be abundant in this genome (Verbruggen 2016). Consistently, homology classification placed 89% of sequences with last common ancestors along the Brachyura lineage, with less than 2% of sequences potentially assigned to other kingdoms (1.8% fungi, < 1% plants, bacteria and viruses). Completeness estimates based on lineage–specific universal eukaryotic marker genes indicate a highly complete genome assembly, with 98.4% of markers detected (Fig. 1c). Most marker genes were assembled at full length and located within large scaffolds. Additionally, the low level of duplicated markers supports the assembly’s high contiguity and suggests minimal structural contamination.

Finally, assessment of the sequence complexity revealed expected high levels of sequences with repetitive nature among the unscaffolded short contigs (Fig. 1d). The presence of repetitive regions in these contigs is consistent with both the classification of a significant fraction of these contigs as putative retrotransposons and the apparent difficulty of scaffolding these contigs unambiguously into a longer scaffold context, thereby contributing to the bimodal sequence length distribution observed.

While our initial draft assembly did not contain a detectable mitochondrial genome, we recovered a single mitochondrial contig of 15,474 bp using a targeted assembly approach. The obtained mitochondrial genome matches those of three brachyuran crabs (*Scylla paramamosain—*JAHFWG010003966.1; *Callinectes sapidus* Rathbun, 1896—NC_012572.1, *Portunus trituberculatus* (Miers, 1876)—NC_005037.1) in size and divergence and appears syntenic in its overall genomic organization (Supplementary Fig. 3).

## Discussion

Here we present a high–quality genome assembly of the green shore crab, *C. maenas*, with a total size of approximately 1.09 Gbp. The assembly is highly complete, containing 98.4% of universal eukaryotic markers, and achieves high contiguity through homology-based scaffolding. This genome fills an important gap within the Brachyura, bringing the total number of publicly available true crab genomes to 11.

Despite its ecological significance as an invasive species and its widespread use as a model organism across multiple research fields, particularly ecotoxicology (Leignel et al. 2014; Rodrigues and Pardal 2014), a complete genome for *C. maenas* was not previously available. Earlier sequencing efforts in 2016 produced a fragmented draft assembly covering only 36% of the estimated genome size, hindered primarily by the genome’s high repetitive content (Verbruggen 2016). Our assembly thus provides a valuable foundation for future studies on the genomic basis of invasion success and supports the species’ utility as a model organism in other lines of research.

Our *C. maenas* specimens were sequenced using an Oxford Nanopore R10.4.1 platform, which offers high-accuracy long reads suitable for de novo genome assembly (Guiglielmoni and Schiffer 2024). We faced recurring pore blockage after approximately 2 h of sequencing. Initially, we suspected that pore blockage was caused by contaminants. To address this, we tested two preservation fluids, optimized DNA extraction protocols, incorporated an InhibitEX step, applied SFR kits and WGA. Despite these efforts, pore blockage persisted, leading us to conclude that secondary structures in the DNA were likely responsible. To maximize data yield and minimize pore blockage, periodic pore scans were performed at 30-min intervals during sequencing. After each run (generally 2 h), a flow cell wash was applied before running the next sample, which helped reopen some of the blocked pores. Similar challenges have been reported in other decapods, such as the tiger prawn (*Penaeus monodon*; Fabricius 1798). The authors also attributed these blockages to the formation of complex secondary structures during sequencing and considered them irreversible (Van Quyen et al. 2020).

To facilitate genomic research in brachyuran crabs and other non–model marine invertebrates, we provide a detailed protocol for long-read sequencing using Oxford Nanopore’s MinION platform, from DNA extraction to strategies bypassing pore blockage. This resource highlights the importance of methodological optimization in generating high–quality genomic data for ecologically and evolutionarily important marine invertebrate taxa.

## Supplementary Material

Supplementary_Figures_1-3_(1)_esaf085

Supplementary_table_1_REV_esaf085

Supplementary_Table_2_esaf085

## Data Availability

Raw sequence data and the assembled, curated genome presented here have been deposited at the European Nucleotide Archive under the project accession PRJEB86588.
