# Supplementary material for: De novo whole genome assembly of the globally invasive green shore crab Carcinus maenas (Linnaeus, 1758) via long-read Oxford Nanopore MinION sequencing
Source: J Hered. 2025 Oct 22;117(3):537–44. doi: 10.1093/jhered/esaf085 (PMC13147170; doi:10.1093/jhered/esaf085)
Supplement: Supplementary_Figures_1-3_(1)_esaf085 [file supplementary_figures_1-3_(1)_esaf085.pdf]

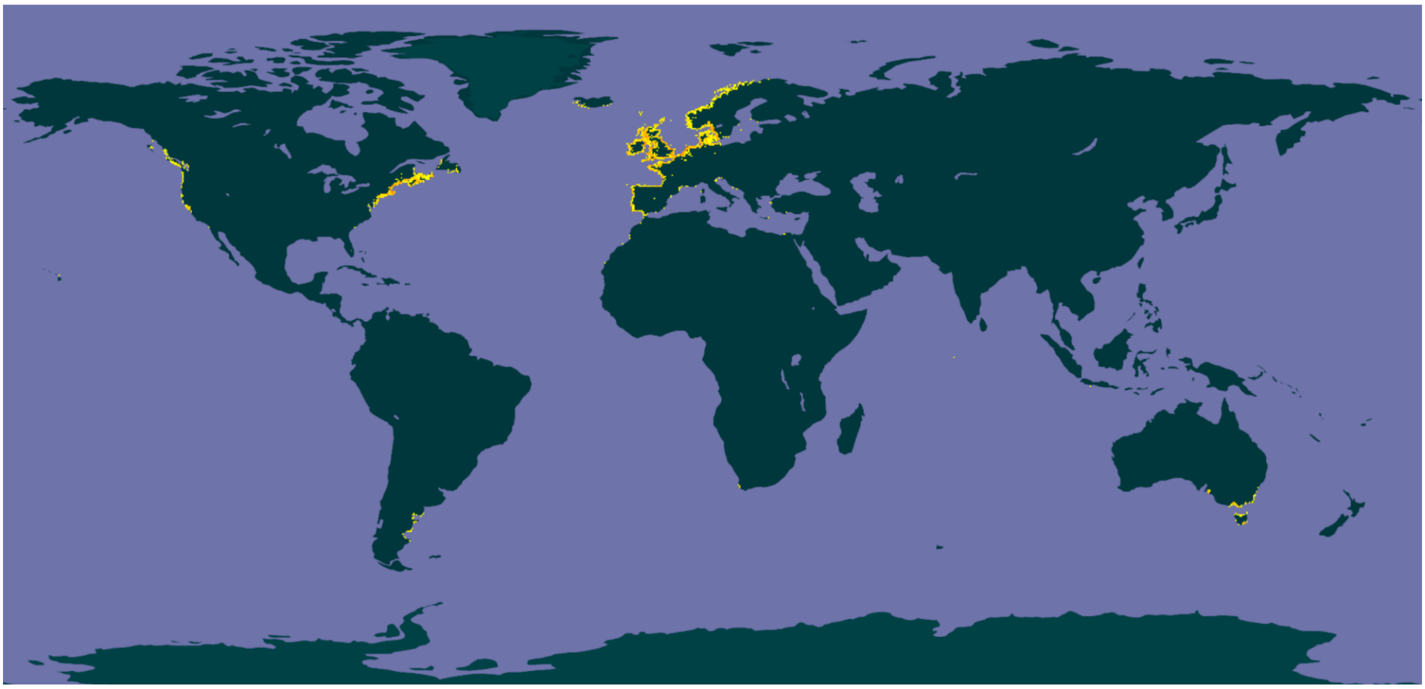

Supplementary Figure 1. Occurrence of *Carcinus maenas* (marked as yellow dots) based on GBIF occurrence data [download December 2024].

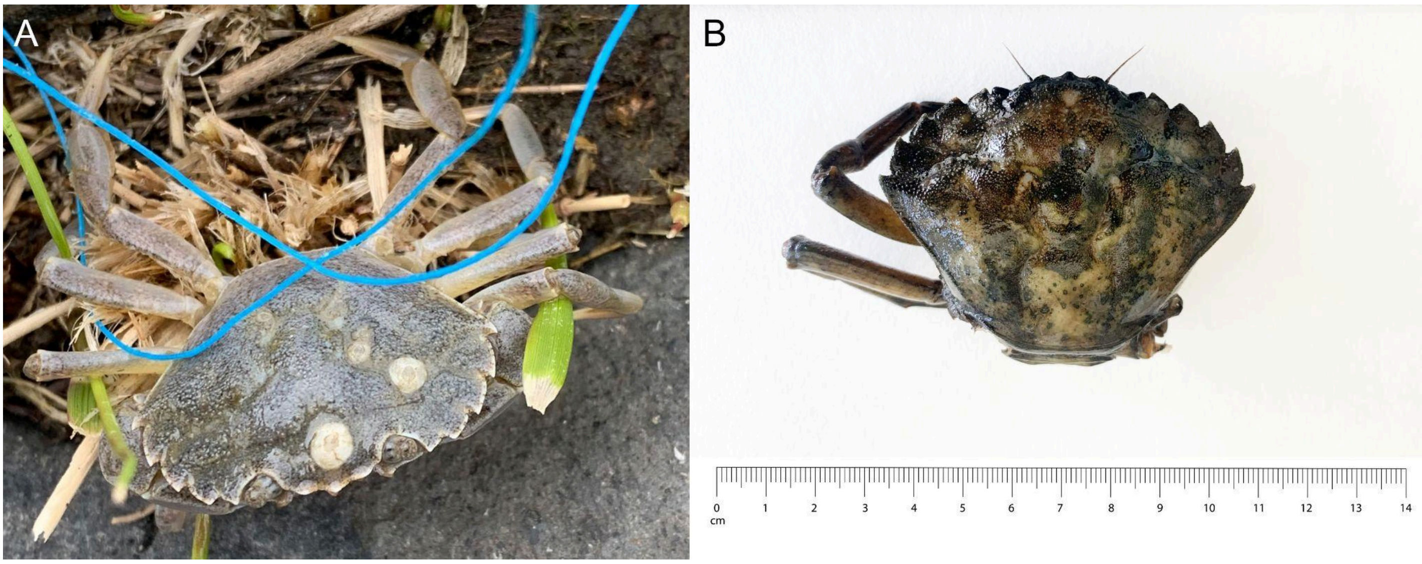

Supplementary Figure 2. A) *Carcinus maenas* specimen collected on 26 April 2023 from the Schiermonnikoog marina, the Netherlands (53.470427, 6.166608), using a line with mussel bait, B) *C. maenas* collected on 11 April 2024 from the harbour of Lauwersoog, the Netherlands (53.410545, 6.208381), specimen was photographed after subsampling.

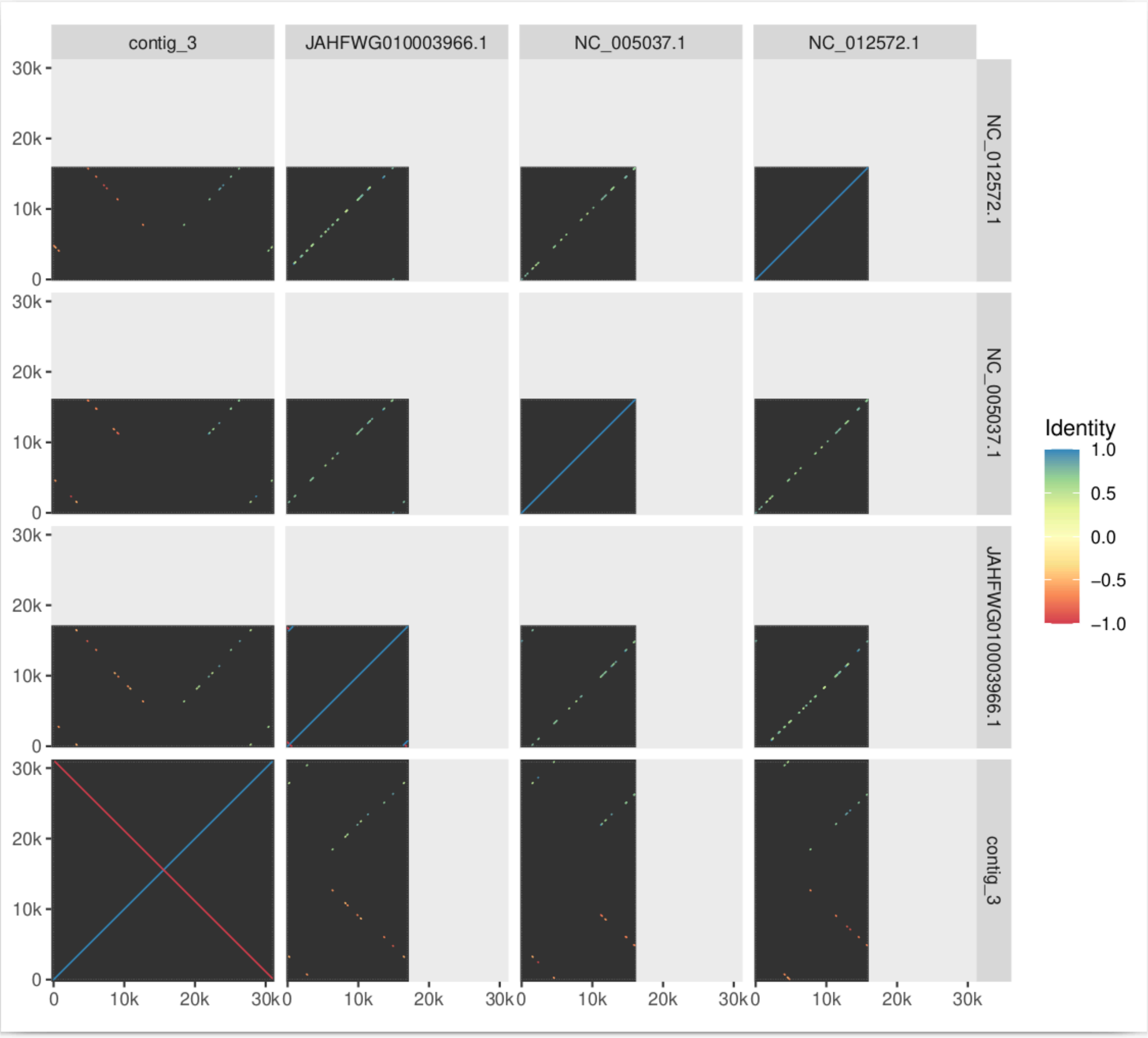

Supplementary Figure 3. Whole genome alignment of mitochondrial candidate contig with close references. Visual representation of all-vs-all whole genome alignments of the three mitochondrial genomes of *Scylla paramamosain* (JAHFWG010003966.1), *Callinectes sapidus* (NC_012572.1), and *Portunus trituberculatus* (NC_005037.1) with the mitochondrial genome candidate contig obtained for *C. maenas* (contig_3) in this study. Regions of sequence similarity between two genomes are indicated by colored lines in the plot, with levels of similarity and direction of the alignment indicated by the color scale (blue: same direction, 100% similarity, yellow: low similarity, red: opposite direction, 100% similarity). The plot shows that the *C. maenas* contig consists of two reverse-complement copies of the actual mitochondrial genome sequence – an assembly artifact caused by the terminal internal inverted repeat structure typically found in mitochondria. This assembly artifact was resolved through manual curation as part of the assembly quality control, and a corrected version of the mitochondrial genome was deposited.
